# The Proposal and Necessity of the Numerical Description of Nano- and Microplastics’ Surfaces (Plastisphere)

**DOI:** 10.3390/polym13142255

**Published:** 2021-07-09

**Authors:** Agnieszka Dąbrowska, Marianna Gniadek, Piotr Machowski

**Affiliations:** 1 Laboratory of Spectroscopy and Intermolecular Interactions, Faculty of Chemistry, University of Warsaw, 1 Pasteura str., 02-093 Warsaw, Poland; 2 Biological and Chemical Research Centre, University of Warsaw, 101 Żwirki i Wigury st., 02-089 Warsaw, Poland; 3 Laboratory of Theory and Applications of Electrodes, Faculty of Chemistry, University of Warsaw, 1 Pasteura str., 02-093 Warsaw, Poland; mgniadek@chem.uw.edu.pl; 4 Horus Sp. z.o.o., 22/8B Bukowińska St., 02-703 Warsaw, Poland; py@piotrmachowski.com

**Keywords:** microplastics, Plastisphere, roughness, quantitative characterization of structures, numerical description, SEM imaging

## Abstract

The constantly growing amount of synthetic materials < 5 mm, called microplastics (MPs), is fragmented in the environment. Thus, their surface, Plastisphere, is substantially increasing forming an entirely new ecological niche. It has already been extensively studied by microbiologists observing the biofilm and by material scientists interested in the weathering of polymer materials. This paper aims to construct a bridge between the physical and chemical description of the Plastisphere and its microbiological and ecological significance. Various algorithms, based on the analysis of pictures obtained by scanning electron microscopy (SEM), are proposed to describe in detail the morphology of naturally weathered polymers. In particular, one can study the size and distribution of fibres in a standard filter, search the synthetic debris for mapping, estimate the grain size distribution, quantitatively characterize the different patterns of degradation for polymer spheres and ghost nets, or calculate the number of pores per surface. The description and visualization of a texture, as well as the classification of different morphologies present on a surface, are indispensable for the comprehensive characterization of weathered polymers found inside animals (e.g., fishes). All these approaches are presented as case studies and discussed within this work.

## 1. Introduction

### 1.1. Microplastics, Nanoplastics and the Plastisphere 

The synthetic materials with at least one linear dimension < 5 mm, called microplastics (MPs), are ubiquitous in the environment [1]. Although they have been extensively studied, the majority of the research is qualitative, and there is a strong need to emphasise the quantitative approach. The MPs behaviour depends on the chemical, physical and biological characteristics, which are directly related to the surface. The transport, fate and adsorption are correlated with the external morphology [2]. For instance, polystyrene (PS), PS-NH_2_ and PS-COOH exhibit diverse dynamic behaviour, aggregation patterns, form structures evolving through time [3]. Functionalization influences the sorption properties by polar and electrostatic interactions, hydrogen bonding, affinity. The nanoparticles of PS had lower sorption capacities of fluoroquinolones than the nano-PS-COOH [4]. As the interfaces determine in large part the properties of materials, their modelling and studies are crucial to understanding the fate of debris. Moreover, due to the fragmentation, the relative surface and ratio surface to the volume is constantly growing to maxima in nanoplastics [5]. The laboratory experiments and theoretical modelling clarify the interaction with organic matter [2]. This expanding new ecological niche was already called the Plastisphere and predicted to have one of the biggest impacts on biota [6]. One can claim it is even the eighth continent. However, regarding the variety of polymer materials and their weathered structures, it seems premature to distinguish just one niche. In the authors’ opinion, one should rather speak of a set of different classes of substrates available for biota [7]. This main hypothesis is already partially confirmed by the microbiological data. Genetic sequencing enables the identification of particular taxa. The types of biofilm vary significantly from one microplastic to another depending, not only on the polymers’ type, but mainly on their structure. Regarding the microbiological data, one can confirm the importance of biofilm research. There is a significant amount of metal [8] and antibiotic-resistant bacteria [9], and this quality is enhanced by the horizontal gene transfer [10,11]. The Plastisphere serves as a new habitat in the vast open ocean of free space [12], especially for the genus *Vibrio*, some photosynthetic filamentous cyanobacteria (*Phormidium*, *Rivularia*), bacillariophyte *Navicula*, *Sellaphora*, *Nitzchia*. The floating and hydrophobic microplastics form a perfect substrate also for numerous diatoms, dinoflagellates (harmful *Alexandrium* included) or other photosynthetic protists. Moreover, they may consist of a vector for transportation of persistent organic pollutants (POPs), algae (also toxic ones), invasive alien species, metal contaminants, etc. To understand properly the state of the surface, one should consider the weathering processes. Synthetic materials were created as durable and persistent, but they are also susceptible to the numerous factors that cause ageing. Naturally occurring fragmentation due to the UV light or mechanical and chemical interactions is further supported by the microbial degradation; for instance, poly(ethylene terephthalate) (PET) decomposed under alkaline conditions had a diameter that diminished from 7.3 to 1.58 µm [13]. The ageing of polycarbonate due to photodegradation leads to a decline in molecular weight and leaching of toxic compounds [14]. The gradual cutting of the carbon chain and formation of reactive oxygen species (singlet oxygen, hydroxyl radical, superoxide anion radical) are caused by the exposition to UV radiation. Active hydrolysis of the hydrocarbon polymer was already confirmed, as well as the decomposition of low-density polyethene (LDPE) [15]. Apart from bacteria inside the biofilm directly adsorbed on a substrate, those in insects’ guts are also responsible for the gradual transformation of synthetic polymers [16]. Cognately, some fungi were also confirmed to biodegrade polymers [17]. Increasing the roughness of the surface fosters the settlement of biota on a Plastisphere. Microplastics have a complex impact on the environment and hundreds of species [18]. Polymer debris is gradually becoming an integrated part of the environment and is propagating via a trophic chain that was reported in various independent research [19,20,21] to have diversified ecotoxicological effects [22]. At the top, humans already experience the presence of microplastics in food, e.g., seafood or fruits and vegetables [23]. It was recently discovered even in the placenta [24]. The issue of microplastics is not limited to the marine environment. For instance, the behaviour and decomposition of particles in soil [25] or freshwater systems [26] are also studied extensively. The polypropylene (PP) might be biodegraded by the Antarctic soil bacteria *Pseudomonas* sp. ADL15 and *Rhodococcus* sp. ADL35. The significant effect on the material was confirmed by the weight loss and the change in Fourier-transform infrared (FTIR) spectra [27]. A more hydrophilic surface is more susceptible to microbial attack. 

All in all, the sampling, monitoring and characterization of micro- and nanoplastics seems to be a vital task. Although numerous methods are used in microplastics’ description [28,29,30,31,32,33], the majority of research is strictly qualitative and simply determines the type of polymer found in the environment. One can list gas chromatography, mass spectrometry, thermal gravimetric analysis [34], optical microscopy, X-ray or a hot needle test. In particular, the spectral characterization of microplastics is already a standard and robust source of information about materials. Although both FTIR and Raman spectroscopy [35] are used as complementary methods, the infrared spatial resolution does not cover the nanoplastics. Raman spectroscopy is more common with fragmented debris [36,37]. To some extent, the micro-FTIR is also useful for smaller fractions of microplastics [38], especially polyamide (PA) fibres with strong self-luminescence in Raman spectroscopy. In all techniques, the proper data and signal pre-processing are crucial [39]. Currently, as the awareness of the impact of morphology on microplastics’ behaviour increases [40], numerous studies are starting to quantitatively describe the polymers’ surfaces. In particular, the roughness, sorption or formation of ecocoronae are studied. The influence of roughness on surface sorption was confirmed, for instance, on polystyrene particles (PS) more subjected to heavy metals (Pb, Cu, Cd, Ni, Zn) when degraded by UV radiation. One recent paper (2020) [41] proposes the usage of atomic force microscopy (AFM) for curvature description, double-shot Pyrolysis Gas Chromatography Mass-Spectrometry for studies of sorption behaviour and Whispering Gallery Mode (WGM) nanosensing for exploring the sorption kinetics. This last parameter is useful in the determination of the environmental impact of adsorbed pollutants, their transport and bioavailability. WGM enables the registration of the internal reflection of light within the microcavities. An evanescent field is generated thanks to the accumulation of photons circulating within the resonator.

This paper addresses the same problems from a different perspective by analysing the pictures from scanning electron microscopy (SEM) to provide numerical data about morphology. The novelty of this approach is related to the reinforcement of the collection of quantitative data that will be important for the classification of the Plastisphere types and, in the future, improve the basic knowledge of this phenomenon. It will also be interesting from the point of view of natural ageing studies. Its main advantage is that SEM pictures are easily available because they are already a standard in research. SEM was also successfully used before for microplastic characterization [42], and it will be discussed more in detail within the next paragraph. 

### 1.2. SEM Pictures as a Base for Image Analysis

Scanning Electron Microscope (SEM) is commonly used in scientific and industrial laboratories for topographical and morphological surface characterization of various materials. It allows study of the topography, morphology, and composition of various solid samples. SEM provides high-resolution images with excellent depth of focus. In SEM, a focused electron beam is scanning the sample surface. While electrons from the beam interact with atoms of the sample, various types of signals are generated and collected by an appropriate detector to create a digital image of the sample surface. The most popular detector collects secondary electrons (SE), which emission depends on the sample’s orientation and morphology. As a result, a high-resolution 2D image is obtained. On the other hand, the amount of generated backscattered electrons (BSE) during the interaction with the primary beam is connected with the sample’s mean atomic number (Z). Backscattered electrons collected as an image by the BSE detector gives high-resolution compositional maps of the 3D sample. The third signal, as important as the two previous ones, is X-ray photons collected by an energy dispersive spectroscopy detector (EDS). In this case, images of spatial variation of chemical composition can be obtained in the form of the elemental distribution map or point/spot elemental analysis. EDS gives both qualitative and quantitative data. 

Microplastics are usually non-conducting materials, and each specimen has an individual morphology that makes them a challenge for non-destructive SEM analysis. A typical workflow for non-conducting samples is covering them with a sputtered conducting layer of metal, alloy, or carbon and imaging with a high energy beam for high-resolution SE micrographs. However, this approach makes samples unusable for further analysis with different techniques (e.g., IR or Raman spectroscopy) and slightly changes the original morphology. Fortunately, as we showed previously [43], imaging and qualitative analysis of non-sputtered MPs is still possible with SEM, working with a low energy beam (0.5–1.5 kV). Many data can be obtained based on images obtained at low energy beams for non-sputtered samples. SEM imaging can provide various information from the simple morphology, and topography changes of the MPs surface, ageing processes control to collecting images for 3D reconstruction and further analysis of surface’s roughness. 

Collecting SEM images for 3D reconstruction in a typical photogrammetry workflow can be time-consuming since it requires collecting a series of images acquired from different angles. A typical SEM device can tilt the sample from −5° to 70°, which in some cases can be insufficient. However, Gontard and co. [44] show how to overcome this problem by acquiring a series of images from positive angles, then plane rotating the sample and acquiring tilted images in the opposite direction. A faster method of 3D reconstruction of SEM images was proposed by Sartipi and co. [45]. The proposed method was based on taking a small number of images using two detectors collecting secondary electrons (SE) and backscattered electrons (BSE) while the sample was rotated. Next, a two-step height optimization procedure was used in the post-processing of the images. A pair of tilted images can be used instead of the AFM [46] to reconstruct the 3D profile.

Various surface morphology and composition have a significant influence on microplastics’ physical properties, biota, and interaction with the environment. Therefore, the assessment of parameters such as roughness seems very important. Atomic force microscopy (AFM) can be used instead of SEM for surface roughness characterization since SEM has limitations in terms of qualitative analysis of obtained images. AFM gives direct 3D information about microplastics surfaces. AFM can work in one of three modes: contact, intermittent contact, and non-contact. In all of the modes, it gives scanning 3D resolution in nanoscale and provides topographical data suitable for surface roughness analysis. However, AFM also has its limitations: it only gives images of a small surface area and the scanning process is very time-consuming. Moreover, mechanical contact between the cantilever tip and the high roughness sample surface can destroy the tip and introduce surface artefacts. To solve this problem two other modes of AFM imaging can be used. To overcome the limitation of both techniques combination of SEM and AFM seems to be a very good solution. SEM provides a large area and high-resolution images while AFM provides spatial resolution [47]. However, within this study, the potential of SEM pictures not complemented by AFM is shown. That is due to the SEM being much more popular among the MPs researchers. Moreover, one wanted to check if it will be sufficient for the first classification of structures and proved its utility. Finally, electron backscattered diffraction (EBSD), laser scanning profilometry or X-ray computer tomography (XCT) are also used for the measurements of the surface topography. The future will be probably dominated by FIB-SEM [48]. After cutting cross-sections the images are taken, the picture analysis is performed. However, this method cannot be considered non-destructive.

### 1.3. The Picture Analyses in Various Research Areas and Different Numerical Approaches

The idea to use images to recover the structural information about a particular material is not new. The analyses of pictures are already implemented and well-developed in the various domains of science, engineering or industry, for instance, to monitor defects, describe layers, characterise interfaces in stretchable electronics [49], fouling and drag reduction in the maritime industry [50], for the modelling of particles behaviour in function of their roughness [51], in porosity control, in situ detection of morphological changes in alloys [52] or steel [53], microcrack propagation [54], etc. There are several approaches and software used for the picture analyses, such as ImageJ. It is possible to extract the pore structure, estimate roughness, discuss the evolution of the cracks, etc. However, within this study, Python is proposed to ensure the open-source tools with a maximum of adjustability. The majority of methods include digital image processing done at the beginning (denoising, data structurization, etc.) and then focus on a particular analysis (e.g., textures, edges, histograms) [55]. SEM pictures may be already statistically described by AI methods [56]. One can use the picture luminosity to recover the structure of surfaces which is a standard approach in material science. Nevertheless, proper 3D visualization requires internal or external calibration. An interesting approach is presented in a work reconstructing the 3D of ferrite-pearlite microstructure by a set of SEM/SE2 images [57]. Finally, one can create the digital material representation by numerical methods, e.g., cellular automata, Monte Carlo, Voronoi tessellation, sphere or ellipsoid packing algorithms. An interesting numerical model of roughness was created for Ti surface in dental implants [58], coal and its micropore structure at the nanoscale [59] or to measure the internal frost damage in types of cement [60]. The real pore microstructure was compared with the fracture simulation. It is also possible to estimate the surface integrity while microcracks are formed [61]. Recently, even the fracture network development was described [62] or the determination of the representative 3D volume element [63]. All those approaches are relevant in the case of microplastics and studying the environmental consequences of their morphological transformations. This paper provides just the first and preliminary results proving the vast spectra of important issues to be explored in further studies.

## 2. Materials and Methods

### 2.1. Microplastics and Nanoplastics Classes and Collection of Samples

One should choose the variety of diversified samples to present the possibilities of numerical picture analysis and the roadmap of the main approaches to the quantitative and qualitative characterization of the Plastisphere. Although the sampling itself is a challenge [64], the standardization of protocols will fortunately not be relevant here. Within this study, a robust set of samples from ghost nets, beaches, seawater, mussels, and fishes (herrings) was selected to provide an overview of the existing marine microplastics. The aim is not to provide a detailed description of ecological issues in all those cases, as already reported, but to show the practical implementation of the Plastisphere characterization on diversified samples, using different approaches. The samples are provided by authors or are from the sources acknowledged at the end of this paper. The majority of analysed debris was from the PP (polypropylene), PE (polyethene) or PS (polystyrene), which corresponds with the most encountered materials. The following crucial issues can be addressed by the quantitative picture analysis numbered in order exactly as the results presented in the subsequent case studies (in Section 3.1, Section 3.2, Section 3.3, Section 3.4, Section 3.5, Section 3.6, Section 3.7 and Section 3.8):

1. the size and distribution of fibres in a standard glass fibre filter (GFF);
2. debris mapping on the filter and their grain distribution;
3. the degradation of fibres (ghost nets);
4. the different patterns of degradation for polymer spheres;
5. number of pores per surface;
6. description and visualisation of a texture;
7. classification of different morphologies present on a surface;
8. comprehensive characterization of weathered polymers found inside animals (e.g., fishes).

All these issues were classified as crucial for the research of microplastics and are presented within this work. This choice aimed to present the easiest way to obtain the relevant quantitative data just from SEM pictures without any additional specialized measurements needed. Our purpose was also to make this overview diversified. 

### 2.2. The Physical and Chemical Qualitative Characterization

Within this study, we used FE-SEM Merlin (Zeiss) equipped with In-Lens and HE-SE2 detectors. Both detectors collect secondary electrons and give good resolution and high-quality images. All measurements were carried out on non-sputtered samples, which enables them for further analysis with different analytical techniques. The lack of coating on a surface is one of the main advantages of this approach. Samples were placed on the aluminium stabs and attached with double-sided carbon sticky tape. Working with non-conducting and non-sputtered samples requires environmental SEM with a low vacuum or special parameters in the case of high vacuum systems (e.g., low electron high tension (EHT) and low sample current). To obtain better quality images we decided to use a combination of signals collected simultaneously by both In-Lens (SE-1) and HE-SE2 (SE-2) detectors. 

For all samples, their polymer origin was previously confirmed by Raman spectroscopy (DXR Raman microscope, Thermo Scientific, CNBCh Warsaw, green line 532 nm) in the preliminary check. The proportion between -CH_2_ and -CH_3_ bands revealed the differences in the level of degradation of particular polymer debris. The roughness caused by weathering was then visualised and analysed. 

### 2.3. The Picture Analysis and the Quantitative Description of Surface

Although one can list numerous software and packages dedicated to picture analysis, within this research Python is proposed. There are several advantages to this solution:

- the user-friendly and fast-developing programming language;
- interactive and standard mode;
- several distributions available (e.g., Anaconda);
- a powerful programming language with clear and elegant syntax making it easy to read and write;
- highly portable, easy to extend;
- an open-source software, with a large community of users.

In all cases, the conventional signal filtering and denoising precedes the data analyses, in particular, edge detection, contours, grain size distribution, histograms, textures, and 3D visualisations. The recalculation size of one pixel to nanometers enables the recovery of a real diameter of structures observed by SEM. The proposed algorithms are freely available on-demand for scientific purposes from the corresponding author. The Anaconda environment was used with the following libraries imported: NumPy, matplotlib.pyplot, cv2, skimage, PIL. 

For the Canny Edge detection, the three parameters are needed: sigma (responsible for the Gaussian Blur), low and high thresholds. All below the low threshold are classified as certainly not the edge, and all above the high threshold are surely the one. The pixels in between are evaluated based on their connection with the main edges. Parameters values are purely subjective, so to obtain the comparable data, the set of those three parameters was fixed once for all series of related images. 

At this stage of our research, the main limitation in the construction of 3D profiles is related to the calibration of the *z*-axis (lack of absolute high values). Thus, the normalized signal was presented. For future work, AFM or a pair of tilted SEMs will resolve this issue. 

The histograms are based on the luminance matrix and sometimes normalized just for practical purposes. Here, the full range 0–255 on a greyscale was preserved. The changes in their behaviour are, in some cases, a good indicator of particular material qualities, providing the same apparatus parameters, which were explained in detail in examples. 

## 3. Results and Discussion

Within this paragraph, the set of obtained results is presented and discussed. One has chosen the examples of particular interest for researchers working with (marine) microplastics—those addressing the main problems encountered during environmental samples.

### 3.1. The Characterization of Glass Fibre Filters (GFF)

**Figure 1:**
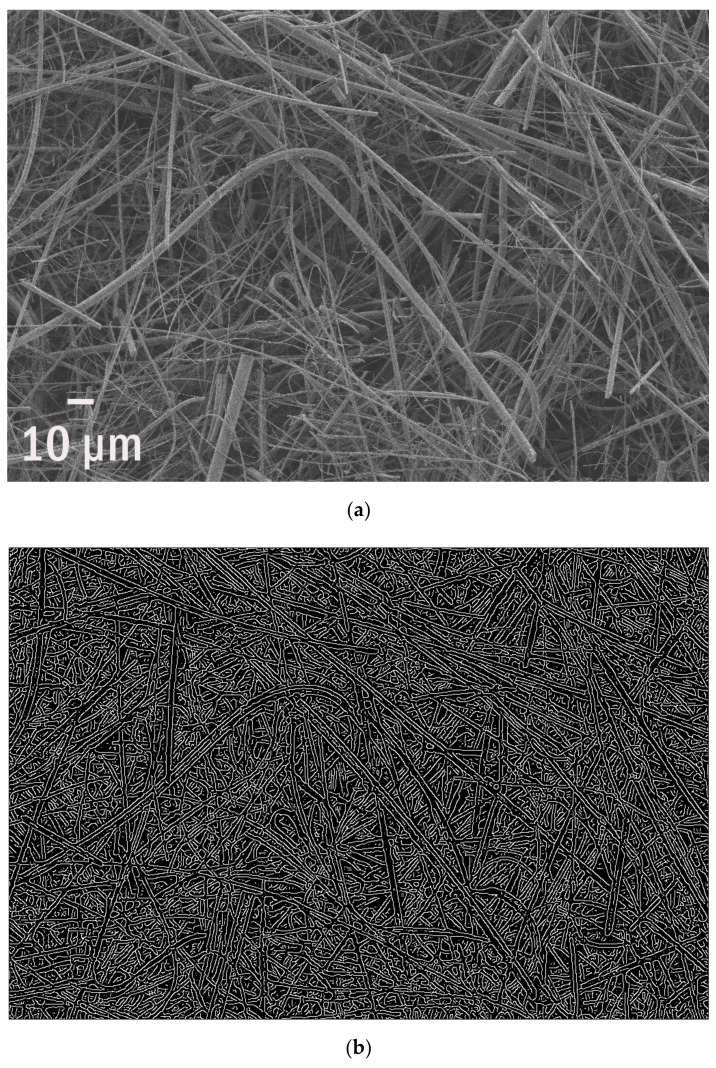
The standard (**a**) glass fibre filter with its random layers and mesh and (**b**) their edges identified.

Glass fibre filters (GFF) are standard in marine microplastics characterization. Despite their drawbacks, such as the self-luminescence in Raman spectroscopy, they are commonly used as cheap, available and efficient. Interestingly, the declared holes’ diameter and fibre size are not so precise and the average distance between them varies approximately in the range from 9 to 12 µm (Figure 1). This information, negligible in the analysis of microplastics, becomes crucial for nanoplastics monitoring where the smallest fraction has to be collected. One can find 720–780 nm of edges’ length per standard area of 1 µm^2^ what was visualized in Figure 1b. It corresponds to the SEM picture (Figure 1a) with a representative fragment of one layer. 

The filter itself is composed of three main fractions of glass fibres with the following real average calculated diameters: 9.5 μm, 5 μm for big and medium, respectively. 

### 3.2. The Detection of Particles and Their Grain Sizes

**Figure 2:**
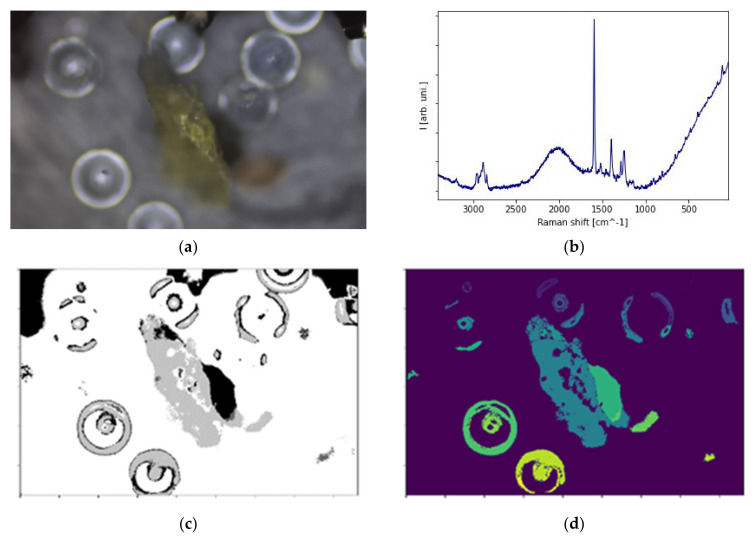

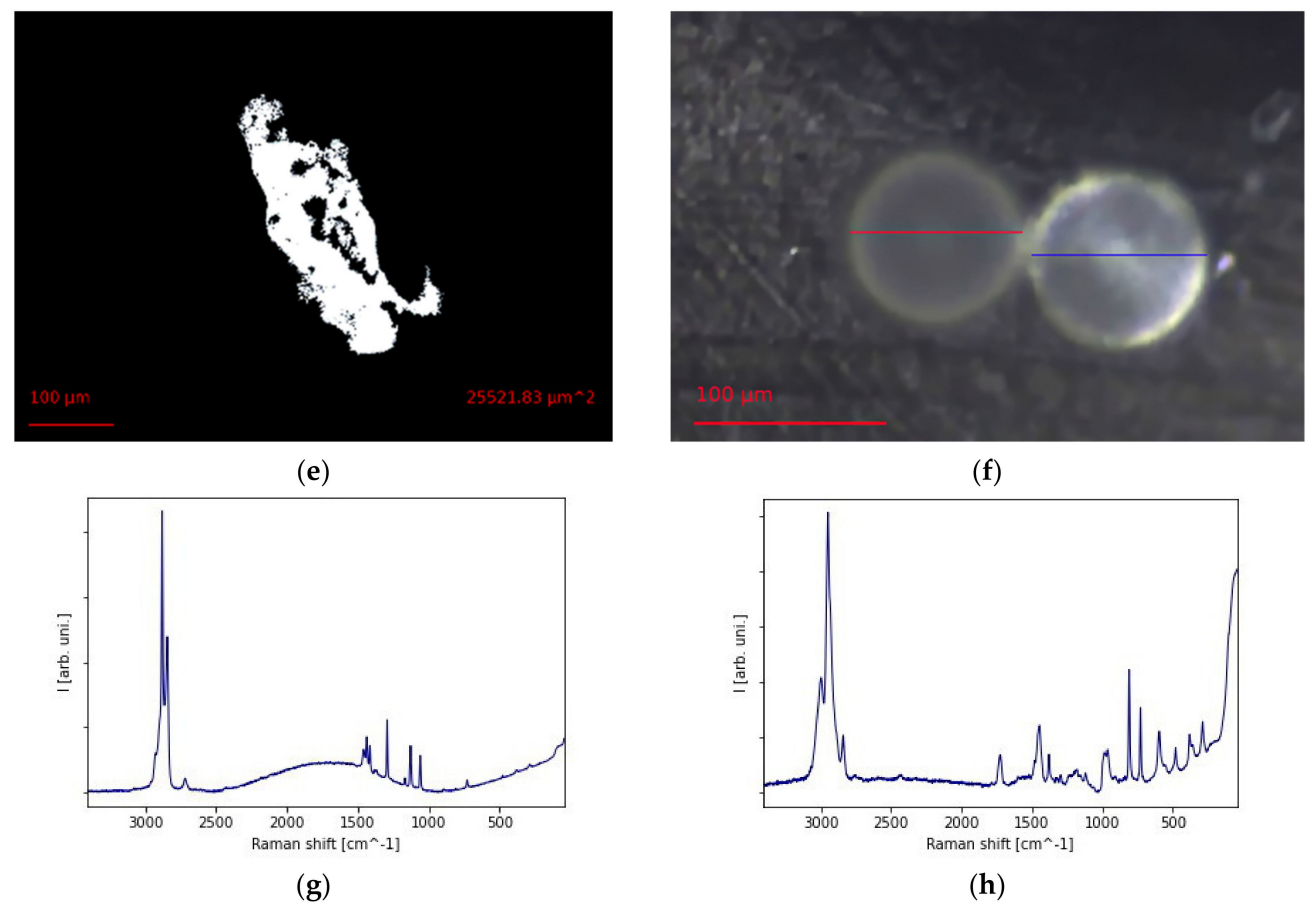
Different debris from a freshwater system mapped on the GFF filter: (**a**) polypropylene (PP) with (**b**) its Raman spectrum and (**c**–**e**) the steps towards object detection and its calculated surface, (**f**) two similar spheres, but identified by Raman spectroscopy as (**g**) polyethylene (PE) and (**h**) poly(methyl methacrylate) (PMMA).

After sampling, the collected material is frequently filtered before the standard Raman spectroscopy measurements. After that, between the numerous objects presented on a filter, one spots the microplastics debris. Instead of a manual search, there are several commercially available packages to automatize this step. The aim is to obtain the easiest, most accessible open-source tool for non-specialists to detect the particles and estimate the grain size distribution. This approach provides information about the environmental context of the found particles; that is the MPs’ size relative to the surrounding fractions of organic and inorganic matter. It is especially useful for non-purified material. The grain size distribution can be estimated without the additional dynamic light scattering (DLS) measurement. However, at this stage, we present only preliminary results: objects detected with the calculated total area, the volume of spherical nurdles based on the radius estimated from picture analysis. All objects were found in the environmental samples from freshwater systems (pristine Lithuanian lakes). Figure 2 presents the three representative different polymer debris found within one sample from sediments and with their polymer origin (PP, PE, PMMA) confirmed by Raman spectroscopy (the characteristic shape of peaks in a C-H stretching bands region). 

Figure 2a shows the aged polypropylene (PP) debris among the spherical nurdles. Its total surface area equals 0.0255 mm^2^. To calculate it from the picture, a three-step protocol was used for detection: converting to the greyscale, thresholding for the edge detection, then labelling (Figure 2b–e). The average volume of different spheres found among the grains on a filter (Figure 2f) was estimated to be ~394,000–418,550 μm^3^. A fast geometrical description provides additional data, such as the diameter, for the identified particles. Here, the apparently similar spheres are, in fact, the two entirely different polymers: polyethylene (PE; Figure 2g) and poly(methyl methacrylate) (PMMA, Figure 2h). Thus, spectral identification is indispensable for proper classification. 

### 3.3. Ghost Netting

**Figure 3:**
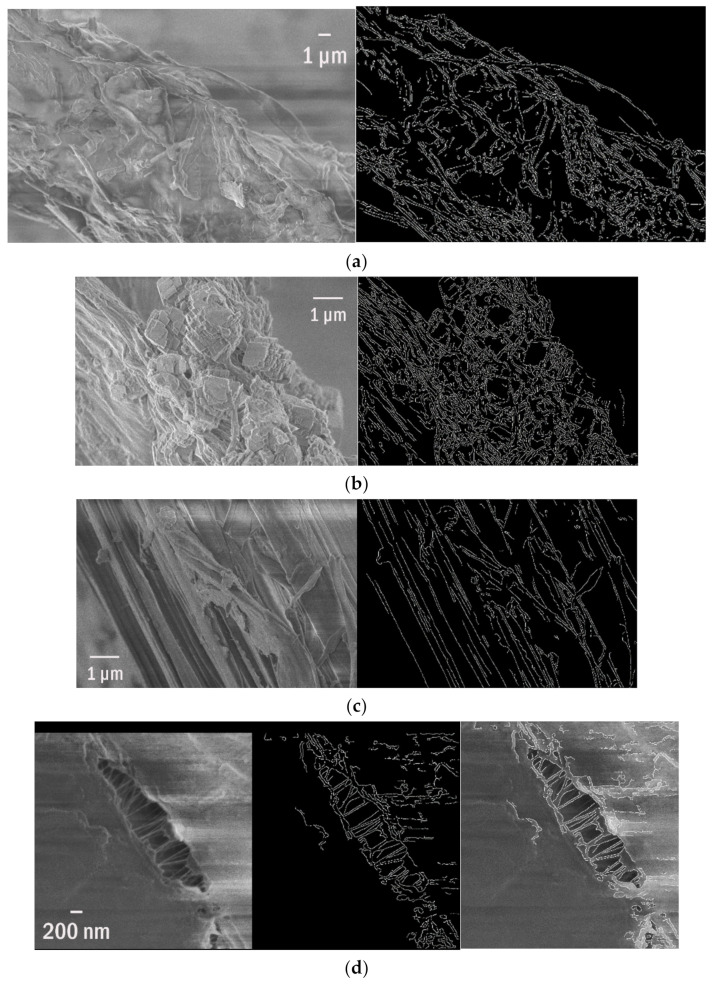
The SEM pictures of (**a**–**c**) three weathered fibres and ghost nets and their edges, (**d**) edge detection of the decrepitude.

The phenomena of ghost nets are one of the main environmental issues of the 21st century. The illegally disposed or lost fishing gear remains in the ocean and creates by-catch or entanglement of fauna [65]. Floating for years, the abandoned nets kill hundreds of animals before their final sedimentation at the seabed [66]. At this stage, they are already with an overlay of the abundant organic matter and naturally aged by UV radiation and saltwater. That makes them a perfect example of the weathering of polymer fibres. SEM pictures reveal numerous cracks, holes, kinks, and adsorbed, accumulated material (Figure 3a–c). Here, they are visualised on the corresponding SEM pictures and parametrized as the length of detected edges. This simple analysis provides an efficient quantitative description of the substrate available for organic or inorganic compounds adsorption or biofilm formation. All ghost nets exhibit significantly enhanced roughness which increases their free surface. The picture analysis enables the description of jaggedness and fibres split for thinner ones with roughness visible. The changes in morphology during the deterioration of fibres, mainly from polypropylene, are observed. The total surface available as a substrate for biofilm increases significantly.

Moreover, one can trace the discontinuity in structure (Figure 3d) and the total increase in surface area due to that. For the deteriorated fragment shown in Figure 3d, the edges are >5 times longer than a perimeter. One can also estimate the length of edges that increases dramatically due to the fibres getting thinner. Finally, traced edges serve as markers for the signs of weathering. This approach also enables the detection of the delamination zones. The spatial resolution is limited only by the SEM quality, not the algorithm itself.

### 3.4. Primary Sources and Nurdles

**Figure 4:**
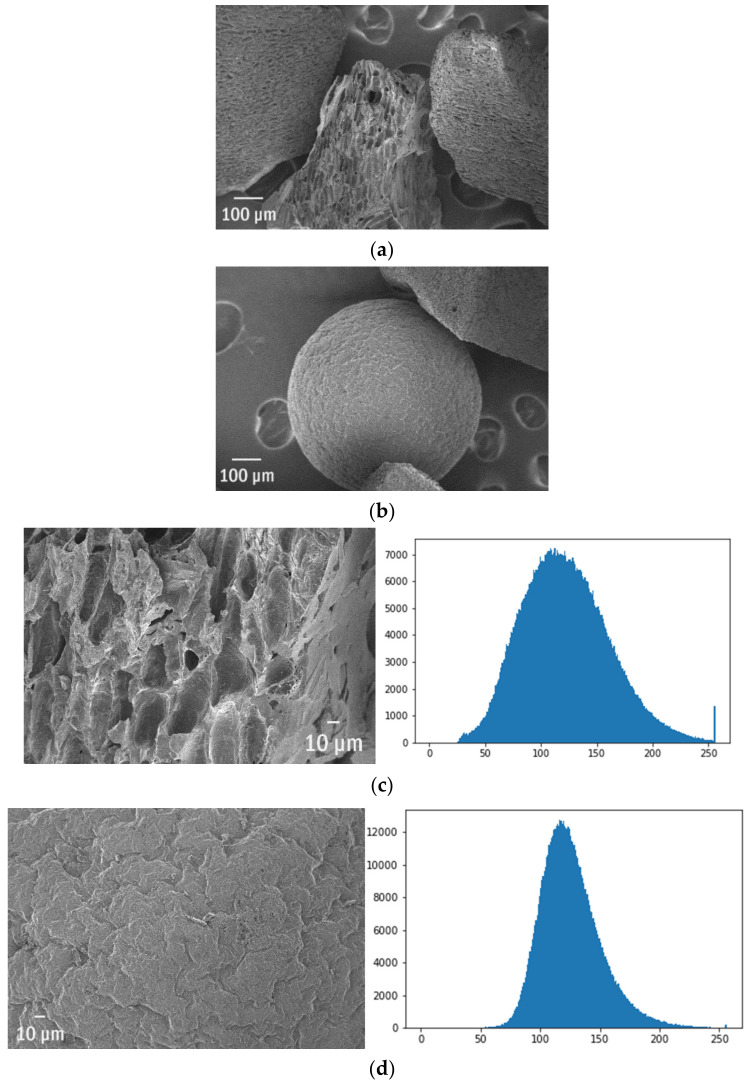
The ageing patterns of two types of microspheres and their SEM histograms (OX—luminosity, OY—number of pixels): (**a**,**b**) the general overview of the microspheres, (**c**,**d**) the two different samples with the same magnitude and their histograms.

Microplastics are commonly divided into the primary and secondary ones depending on if they were delivered to the environment already being <5 mm or gradually fragmented after the disposal. The primary sources [67] are mainly related to the particles in household chemicals or cosmetics, e.g., scrubs and peelings [68,69,70], or fibres washed out from synthetic textiles. Other important sources are nurdles or microspheres. This example deals with two types of polymer microspheres that after the natural weathering exhibit entirely different surface morphology (Figure 4a,b). The less homogenous and more rough materials have more edges that accumulate and scatter electrons, so under the same measurement parameters, the broadening of the histogram is observed (Figure 4c,d). Thus, the shape of the histogram may be the first quantitative indicator of ageing.

Figure 4c presents the magnified piece of the first sphere (from Figure 4a) and the corresponding histogram. In the same way, one can observe the less worn surface of a second sphere (from Figure 4b) characterized by the narrower histogram. The ideally flat surface, without any cracks or crashes, will have a narrow histogram due to the constant luminescence. Interestingly, those nearly identical, two primary microplastics exhibited extremely different ageing behaviour. It is crucial to provide microspheres that have lower roughness and therefore create fewer chances for instance for POPs or toxins adsorption. More porous structures are better substrates for adsorption. The analysis of the histogram is a first and fast inspection of the surface roughness and may provide a numerical indication for the probability of adsorption on particular debris.

### 3.5. Natural Weathering of Polymers on a Seacoast 

**Figure 5:**
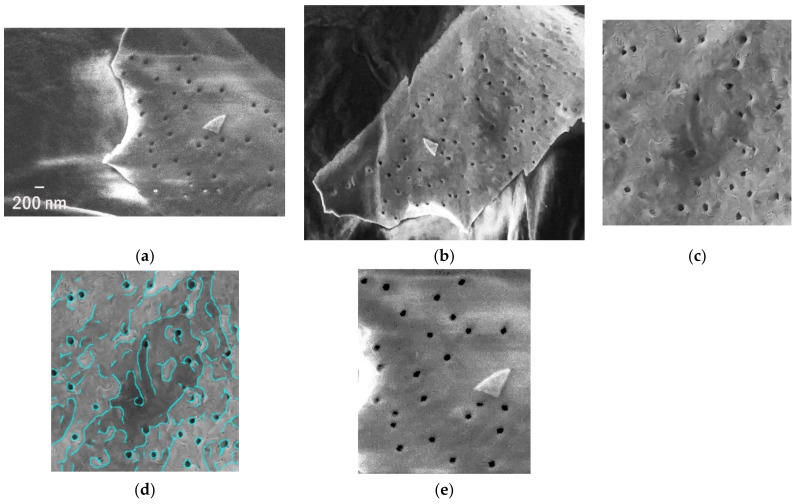
The MPs from Southern Baltic and its perforation: (**a**,**b**) the fragment and whole debris, (**c**,**d**) the magnified piece of debris and marked detected edges, (**e**) 25 holes mapped on a determined area of 6 µm^2^.

Polymer materials, although durable and persistent, are not indestructible and exhibit various traces of natural impact on their surfaces. Sometimes, those patterns are intriguingly regular (Figure 5a). In this example, the specimen, the microplastic debris from the Southern Baltic (the Polish seacoast), was exposed to natural weathering conditions, such as UV radiation and mechanical abrasion, in the breaking wave zone. The holes created on its surface are the ideal habitat for settlement (Figure 5b). 

They covered, on average, 0.9–1.8% of the total area (as detected in Figure 5c), and their surface density was estimated to be approximately 3.5–4.2 holes per µm^2^, ~4 µm^2^ each (Figure 5e). The perforation of polymers is one of the most frequent changes in morphology observed for microplastics found on the seacoast. Usually, it appears simultaneously with the leakage of added compounds. The microplastics pollution in the Southern Baltic is ubiquitous and includes the beach, water and sediments [71]. 

### 3.6. Natural Textures and Patterns

**Figure 6:**
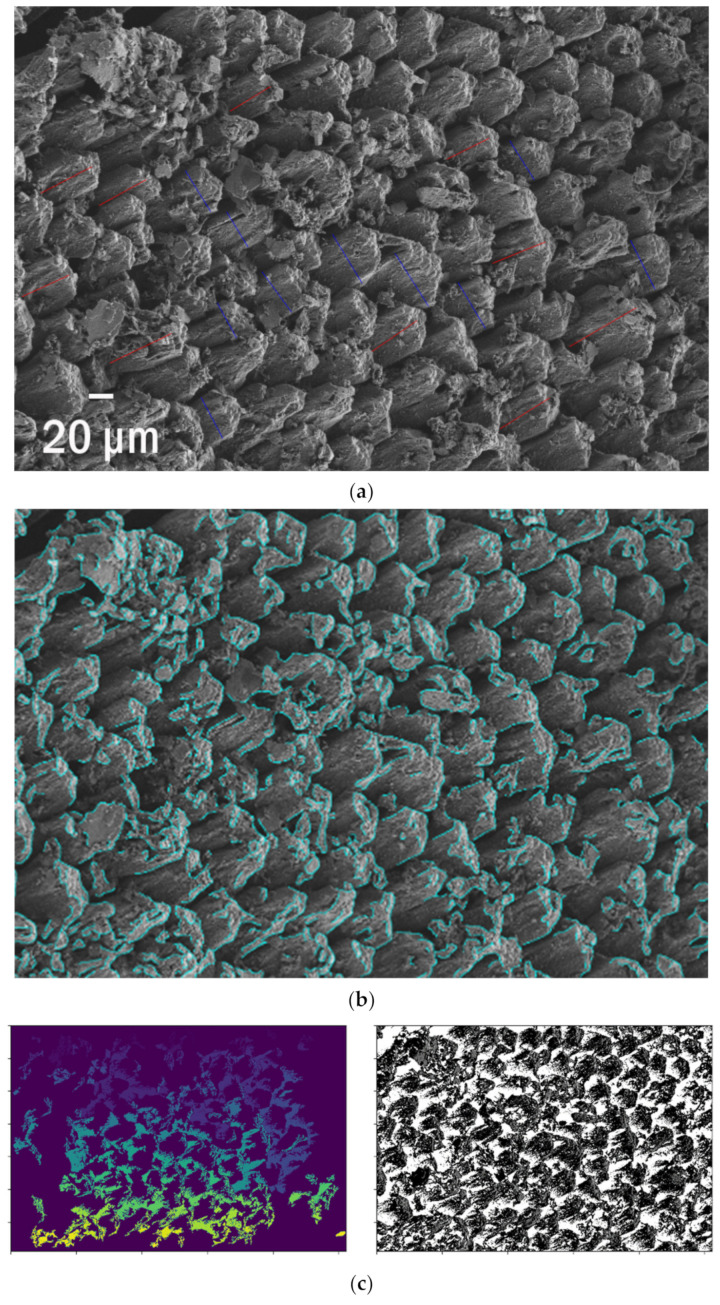
The regular texture of (**a**) naturally weathered material found in the animal’s guts and contours of repeatable “horns” forming its structure, (**b**) tracked edges, (**c**) possible visualization of geometric patterns.

The presence of regular structures, as in a previous example, is not an exception. The piece of foil from guts had this regular pattern and a repeatable texture (Figure 6a). 

The basic concept from picture analysis of pixel or voxel can be used to model such structures as a sequence of transposed basic elements (the “original puzzle”). It is enough to determine this basic unit to recover all morphology. The rough instead of plain structure results in the bigger surface available. For instance, the length of the total edges (1.13 mm), as found in Figure 6b, was estimated to be over 10 times higher (11.9) than the perimeter of a chosen fragment. It results in 2 µm of edges for the area of 1 µm^2^. The average height of one “horn” was estimated to be ~2.069 μm and width ~1.761 μm. 

Figure 6c shows a set of results for a couple of simple approaches to the picture analysis done to obtain the clear geometrical visualization of a pattern and a possible clustering of structures. 

### 3.7. The Variety of Substrates within One Debris

**Figure 7:**
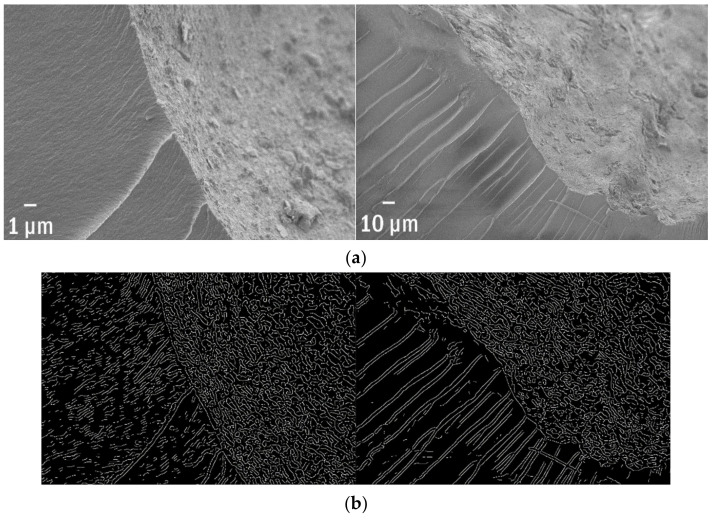
An aged green polypropylene formed at least (**a**) four different ecological niches on its surface, which are easily distinguishable by the (**b**) edges’ patterns.

The ageing processes do not need to be homogenous on all surfaces, and usually one may observe the adjoining of different zones. It is related, for instance, to the different exposure to UV radiation. In that case, picture analysis enables the fast recognition of various types of structures and concurrent classification of the different niches. Both pictures (Figure 7a) present the four different substrates to compare them together.

Although in the proposed example the different areas are visible to the naked eyes, it does not necessarily need to be so. Sometimes differences are more subtle, but still significant from an ecological point of view. The edge pattern, properly calibrated, might be a quantitative metric for the classification of neighbour niches. In Figure 7b, one may observe in detail the differences. The four zones differ significantly in the total length of edges per perimeter (Table 1), which corresponds with a good correlation to their visual characteristics.

### 3.8. Microplastics Detected Inside the Comestible Fishes 

**Figure 8:**
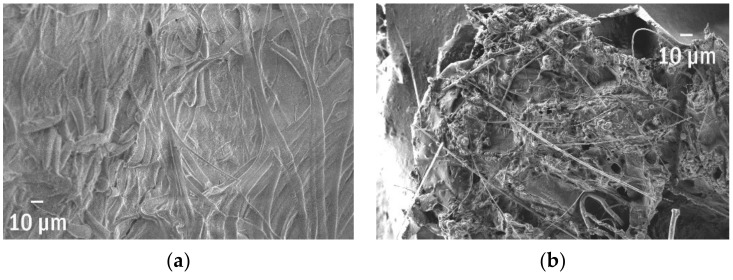
The microplastics (from fish guts of (**a**) cod (the close view of the surface) and (**b**) herring, respectively) and their clear signs of ageing.

The increasing amount of marine microplastics cause their presence in the environment and the rapidly increasing number of items found in fish guts [72]. This case deals with the polymer particles found inside two comestible fishes from the Southern Baltic region—cod (*Gadus morhua callarias*) and herring (*Clupea harengus membras*)—within the ecological study described elsewhere. All specimens revealed clear signs of degradation (Figure 8): cracks, wrinkles, indentations, while the pristine materials have plain and smooth surfaces. 

**Figure 9:**
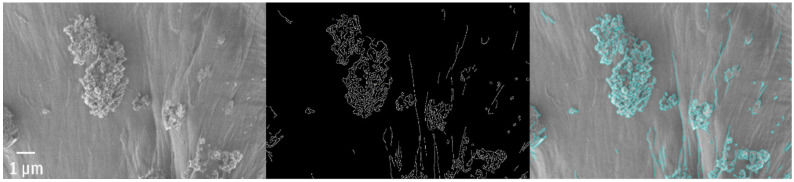
The rubbish bag with adsorbed material and the length of the total edge ~217.5 µm.

**Figure 10:**
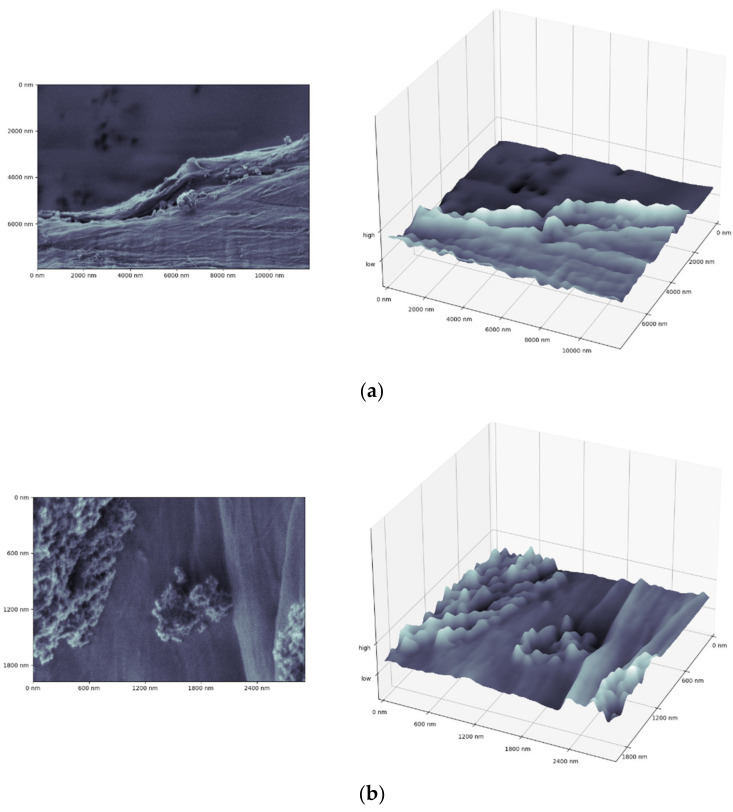
The 3D reconstruction of (**a**) the rough rubbish bag surface with (**b**) adsorbed elements (specimen from the fish’s guts).

Among the most spectacular object found in fishes, one can list the fragment of a bottle tap or a waste bag. The rubbish bag foil cracked and was covered by numerous adsorbed particles that extensively enlarged its surface and the length of edges (Figure 9). The length of total edges, shown in Figure 9, superated >5.5 times the perimeter and reached 2.3 µm per 1 µm^2^. Moreover, it is possible to visualize the surface in 3D as presented in Figure 10 for the fragment of the foil (probably a rubbish bag) found inside the guts. 

The total surface estimation can be accurate only by providing the information on the *z*-axis and thus is not discussed here. 

## 4. Conclusions and Future Perspectives

In the years to come, it seems that the Plastisphere will be gradually expanding and diversifying its ecological niche. Therefore, it is crucial to better understand and classify its surface, providing the most detailed and accurate numerical description. Within this research, this perspective is drawn. This holistic approach is necessary to better understand the emerging eighth continent and its variety of niches. The most up-to-date research has confirmed the complex bacterial interactions on MPs [73]. Some advanced studies even into details considering colonization patterns, taxonomy and metabolism [74]. However, the qualitative and quantitative methods of surface characterization are indispensable to properly classify the different types of particles and find possible correlations between their morphology and the ecological phenomena. One can underline the importance of this emerging research area considering, for instance, the amount of antibiotic-resistant bacteria already found on a Plastisphere. To the authors’ best knowledge, these are the first preliminary results that head in this direction—to provide the numerical parameters for the MPs that will be correlated with the expanding microbiological data. They are also compatible with the source-pathways-receptors model described recently [75]. Certainly, the presented strategies need to be developed, for instance, by better noise-signal separation, deconvolution of the signal and apparatus function [76] or calibrating the 3D profiles to calculate the total surface. Parameters, such as roughness, fractal dimension [77] and lacunarity, are also recommended as the next step and an efficient approach towards an unequivocal quantitative characterization. Although limited, the obtained results indicated that even an attempt to extract the comprehensive parameter may open a new perspective. One starts to classify objects to describe them numerically and in a reliable, reproducible way. At this stage, the main limitations are due to no final adjustments for pre-processing parameters (Gaussian blur, threshold values in edges detection, the contrast of images, etc.). The future research will go in the direction to eliminate to a reasonable extent the subjective selection of pictures to be analysed. Furthermore, one can create a set of parameters comprehensively characterizing morphology. Only by providing that can the correlation with microbiological patterns be obtained.

Apart from the SEM measurements, the important data can be collected via non-destructive advanced methods, such as X-ray diffraction contrast tomography and near-field high-energy X-ray diffraction microscopy (nf-HEDM). Furthermore, deep learning and artificial intelligence (AI) algorithms [78] are the future in studies searching for the correlation between biofilm and structures, exactly as has already happened in MPs spectroscopy [79]. However, the proper data sets are crucial to train AI algorithms. For that reason, the correlation of numerical parameters with the microbiological data will be the bottleneck. With the bacteria sizes ranging between 200 nm and 600 µm, not all morphological changes seem important. However, even if below the biofilm size, the available surface and its structure determine adsorption, transport, sedimentation and the fate of particular debris. All those conditions are of vital meaning for microbial settlement. Finally, one should underline that the picture analyses do not exclude, or even claim to exclude, the detailed physical and chemical characterization of micro- and nanoplastics by, for instance, FTIR and Raman spectroscopy or PCR sequencing, to describe the biofilm. It is a complementary approach that in all probability will be substantially developed during the years to come as proved by the first emerging papers correlating some physical, chemical and morphological aspects with the biofilm [80].

## Figures and Tables

**Table 1:** Characterization of niches by edges’ length.

SEM of a Plastisphere	% of Edges	Edge Per 1 µm^2^	Edge/Perimeter
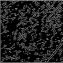	6.65 %	2.394 µm	6.80
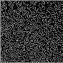	10.96 %	3.944 µm	11.20
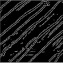	10.84 %	204.25 nm	5.80
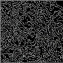	5.67 %	390.41 nm	11.09

## Data Availability

The data presented in this study are available on request from the corresponding author.
